# Traumatic myiasis in wild animals admitted to a rehabilitation center in southeastern Turkey

**DOI:** 10.3389/fvets.2026.1892985

**Published:** 2026-10-06

**Authors:** Ayse Ekinci Yildiz, Duygu Neval Sayin İpek, Aysun Ekinci, Fırat Aşır

**Affiliations:** 1 Dicle Wild Animals Protection and Rehabilitation Practice and Research Center, Dicle University, Diyarbakir, Türkiye; 2 Department of Parasitology, Faculty of Veterinary Medicine, Dicle University, Diyarbakir, Türkiye; 3 Department of Biochemistry, Faculty of Medicine, Dicle University, Diyarbakir, Türkiye; 4 Department of Histology and Embryology, Faculty of Medicine, Dicle University, Diyarbakir, Türkiye

**Keywords:** COI barcoding, dipteran larvae, molecular identification, traumatic myiasis, wildlife, wildlife rehabilitation

## Abstract

**Background and objectives:**

Traumatic myiasis develops when dipteran larvae colonize pre-existing wounds and may complicate the management of injured wildlife. Wildlife-associated cases remain insufficiently documented, particularly in reptiles and wild ungulates. This study aimed to characterize traumatic myiasis in wild animals admitted to a rehabilitation center in southeastern Turkey and to identify the causative dipteran species using integrated morphological and molecular approaches.

**Materials and methods:**

Eight wild animals with traumatic myiasis admitted between December 2020 and September 2022 were evaluated. Larvae collected during routine wound management were identified morphologically. Forty third-instar larvae, five from each clinical case, underwent mitochondrial cytochrome c oxidase subunit I (COI) sequencing. Following quality control and within-case redundancy assessment, 11 representative study sequences were retained for comparative analyses. Species assignments were evaluated using sequence similarity, pairwise nucleotide divergence, and maximum-likelihood phylogenetic analysis.

**Results:**

A total of 132 third-instar larvae were recovered. Four dipteran species were identified: *Lucilia sericata, Calliphora vomitoria, Calliphora vicina,* and *Wohlfahrtia magnifica*. *L. sericata* was detected in six cases, and two common buzzards had mixed-species infestations. The 11 representative study sequences showed 99.52–100.00% identity to verified GenBank references and were deposited under accession numbers OR642793–OR642803. The final 624-bp phylogenetic dataset comprised 16 sequences (11 study sequences, four species-specific references, and one outgroup) and supported the morphological assignments. Traumatic myiasis was also documented in *Rafetus euphraticus* and *Capra aegagrus*, representing potentially novel or rarely documented host associations.

**Conclusion:**

Combined morphological examination and COI-based molecular analysis provided concordant species-level identification of myiasis-causing larvae in this small opportunistic wildlife case series. The findings expand descriptive knowledge of traumatic myiasis in wildlife from southeastern Turkey.

## 1 Introduction

Traumatic myiasis is a parasitic infestation caused by dipteran larvae developing in pre-existing wounds of vertebrate hosts and is considered an important veterinary and medical condition worldwide (1, 2). Larval infestation may lead to progressive tissue destruction, delayed wound healing, secondary bacterial infection, and, in severe cases, death, particularly in debilitated or injured animals (1, 2). Although traumatic myiasis has been extensively investigated in domestic animals and humans, reports involving wildlife species remain relatively limited (3, 4). Recent case reports and reviews nevertheless indicate that myiasis can affect a broad range of wild and captive avian, mammalian, and reptilian hosts, particularly when traumatic wounds or impaired condition are present (5). In free-ranging wildlife, traumatic injuries, impaired mobility, malnutrition, and reduced grooming ability may facilitate larval colonization and increase susceptibility to infestation (6).

Among the dipteran species associated with traumatic myiasis, members of the families *Calliphoridae* and *Sarcophagidae* are of particular veterinary importance (7). Species such as *Lucilia sericata*, *Calliphora vicina*, *Calliphora vomitoria*, and *Wohlfahrtia magnifica* have been reported as causative agents of wound myiasis in domestic animals, humans, and occasionally wildlife species (8–11). *L. sericata* is recognized as one of the most opportunistic and widespread facultative myiasis agents, whereas *W. magnifica* is an obligate parasite capable of causing severe and rapidly progressive tissue damage (11, 12). Previous studies have documented traumatic myiasis caused by *L.* spp. and *C.* spp. in wild birds and reptiles; however, available data regarding wildlife hosts remain fragmentary and geographically limited (5, 12, 13).

Accurate identification of myiasis-causing larvae is essential for understanding host associations and documenting species diversity in wildlife-associated infestations. Conventional morphological examination remains the standard method for species identification; however, morphological evaluation may be challenging in damaged specimens or immature larval stages (14, 15). Therefore, molecular approaches based on mitochondrial cytochrome c oxidase subunit I (COI) gene sequencing have become increasingly important for reliable species-level identification and confirmation of dipteran larvae (16). The integration of morphological and molecular techniques also improves the accuracy of comparative analyses among geographically distinct isolates and contributes to reference sequence databases for myiasis-causing species (16, 17).

Southeastern Turkey represents an ecologically important transition zone supporting diverse wildlife and seasonal movements of migratory birds. Diyarbakır and its surrounding areas also harbor several myiasis-associated calliphorid species. Previous entomological surveillance in Diyarbakır documented *L. sericata*, *C. vicina*, and *C. vomitoria* and demonstrated marked seasonal variation in their activity, indicating that local environmental conditions may influence opportunities for wound colonization (18).

Despite increasing awareness of traumatic myiasis in wildlife, information regarding the occurrence and molecular characterization of myiasis-causing dipteran species in injured wild animals from southeastern Turkey remains limited. In addition, traumatic myiasis in the Euphrates soft-shelled turtle (*Rafetus euphraticus*) and wild goat (*Capra aegagrus*) appears to be rarely documented, and potentially previously unreported, based on the available literature. Therefore, this study aimed to characterize traumatic myiasis cases detected in wild animals admitted with traumatic wounds to the Dicle University Wildlife Protection, Rehabilitation, Practice and Research Center between 2020 and 2022 and to identify the associated dipteran species using combined morphological and COI-based molecular methods.

## 2 Materials and methods

### 2.1 Ethical considerations and animal welfare

The study was conducted under the official scientific research authorization issued by the General Directorate of Nature Conservation and National Parks, Ministry of Agriculture and Forestry of the Republic of Turkey (Decision No. E-21264211-288.04-8920478, dated 16 February 2023). The authorization permitted research on wild animals admitted to the Dicle University Wildlife Protection, Rehabilitation, Practice and Research Center for clinical care and rehabilitation, without additional field sampling.

All animals included in the study had been admitted to the Center for routine clinical treatment. Wildlife examinations and sample collection were performed within the scope of the above authorization. Larval specimens were collected only during clinically indicated wound cleaning and management procedures, and no additional intervention was performed for research purposes beyond standard veterinary care. All procedures were carried out in accordance with applicable national regulations and institutional standards for the care and welfare of wild animals.

### 2.2 Study area and study period

This study was conducted at the Dicle University Wildlife Protection, Rehabilitation, Practice and Research Center in Diyarbakır, southeastern Turkey (37°55′N, 40°14′E, altitude approximately 670 m), between December 2020 and September 2022. Diyarbakır is located in southeastern Anatolia and is characterized by a continental climate, with hot, dry summers and relatively cool, wetter winters. The geographical location of the study area is shown in Figure 1.

**Figure 1:**
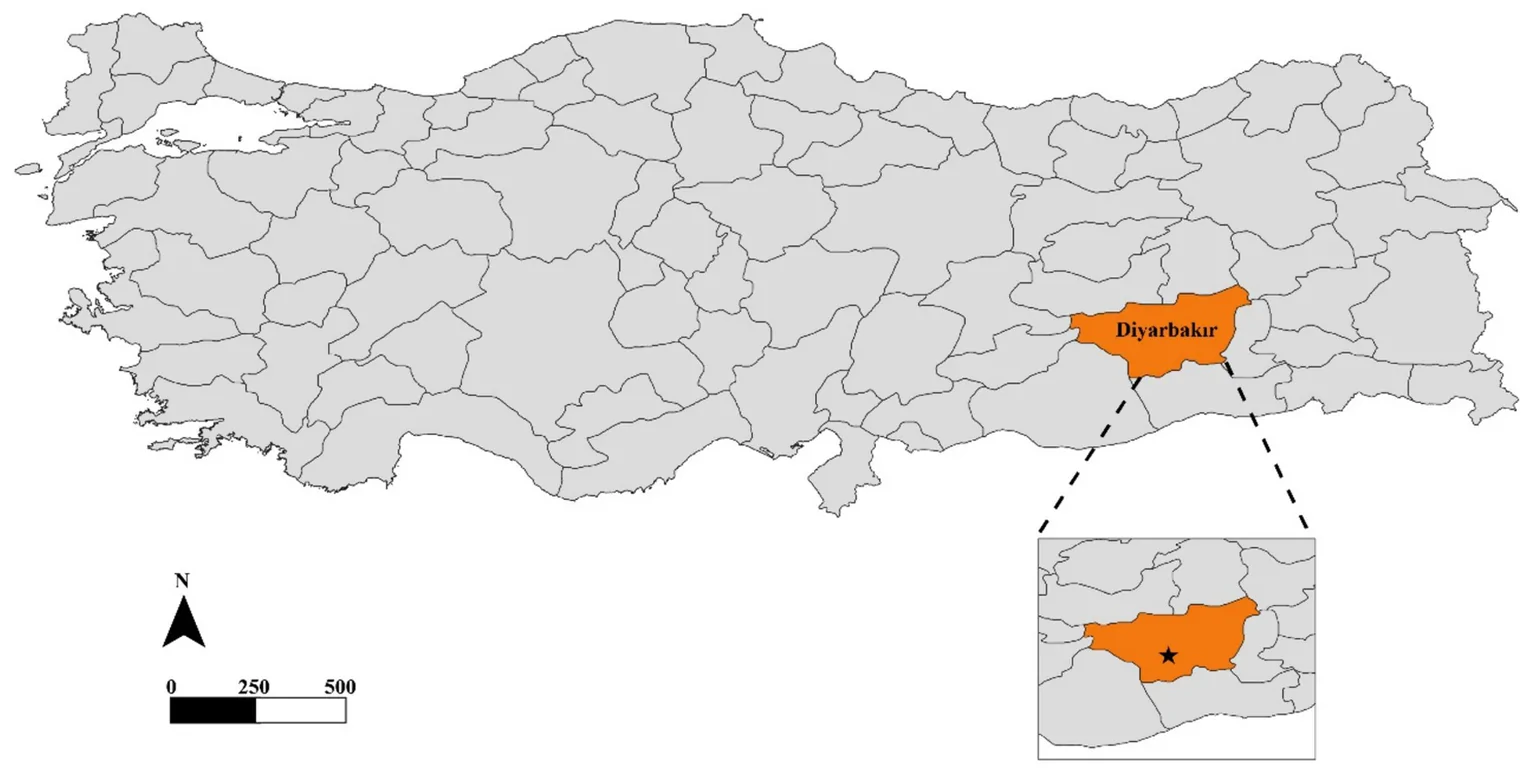
Geographical location of the Dicle University Wildlife Protection, Rehabilitation, Practice and Research Center in Diyarbakır, southeastern Turkey.

### 2.3 Animals, clinical examination, and larval collection

Eight wild animals admitted to the Dicle University Wildlife Protection, Rehabilitation, Practice and Research Center with traumatic injuries and diagnosed with traumatic myiasis between December 2020 and September 2022 were included in the study. Case-specific host, clinical, and outcome information is provided in Table 1. The tortoise could not be reliably identified to species level from the available clinical records and morphological information and was therefore conservatively reported at the family level (*Testudinidae*).

**Table 1:** Clinical characteristics, larval burden, molecular identification, and clinical outcomes of wild animals diagnosed with traumatic myiasis.

Case	Animal species	Month of admission	Clinical finding/wound location	Total larvae	Larvae used for molecular analysis	Identified larval species	Study GenBank accession no.	Reference accession no.	Identity (%)	Clinical outcome
1	*B. buteo*	December 2020	Open wound on the wing	8	5	*L. sericata*	OR642797	DQ345086.1	100.00	Recovered clinically but remained under long-term rehabilitation because of permanent wing damage
2	*B. buteo*	April 2021	Open wound on the wing	6	5	*L. sericata*	OR642795	DQ345086.1	99.69	Recovered clinically but remained under long-term rehabilitation because of permanent wing damage
3	*B. buteo*	June 2021	Open wound on the wing	5	5	*C. vicina* + *C. vomitoria*	OR642801 (*C. vicina*); OR642802 (*C. vomitoria*)	MW266392.1; NC_028411.1	99.70; 99.85	Recovered clinically but remained under long-term rehabilitation because of permanent wing damage
4	*B. buteo*	July 2021	Open wound on the wing	7	5	*L. sericata* + *C. vomitoria*	OR642800 (*L. sericata*); OR642794 (*C. vomitoria*)	DQ345086.1; NC_028411.1	100.00; 99.85	Recovered clinically but remained under long-term rehabilitation because of permanent wing damage
5	*C. ciconia*	August 2021	Open wound on the wing	10	5	*L. sericata*	OR642793	DQ345086.1	100.00	Clinical improvement was achieved, but irreversible wing injury prevented release; the bird remained under rehabilitation
6	*Testudinidae* (species not determined)	May 2022	Open wound under shell	16	5	*L. sericata*	OR642799	DQ345086.1	99.85	Successfully rehabilitated and released into its natural habitat following complete clinical recovery
7	*C. aegagrus*	July 2022	Open wound on the right gluteal region	38	5	*W. magnifica*	OR642803	MT027910.1	99.52	Despite intensive treatment and supportive care, the animal died because of progressive deterioration of its general condition
8	*R. euphraticus*	September 2022	Open wound under shell	42	5	*L. sericata*	OR642796; OR642798	DQ345086.1	99.85; 100.00	Successfully rehabilitated and released into its natural habitat following complete clinical recovery

Molecular identity values represent nucleotide sequence identity between study COI sequences and the verified GenBank reference sequences used in the revised molecular analysis. Five larvae from each clinical case were subjected to molecular analysis. In mixed infestations, molecular sequencing confirmed the presence of both morphologically identified species. Study sequences were deposited in GenBank under accession numbers OR642793–OR642803.

At admission, each animal underwent clinical examination, and the location and characteristics of the traumatic lesion, presence of larval infestation, treatment procedures, and clinical outcome were recorded. Before wound treatment, visible larvae were carefully removed using sterile forceps. The total number of larvae recovered from each animal was recorded, and the specimens were transferred to individually labeled containers containing 70% ethanol until morphological and molecular examination. Separate sterile instruments were used for each animal to minimize the risk of cross-contamination among specimens.

Following larval removal, wounds were mechanically cleaned and treated with an antiseptic solution. A 1% doramectin preparation (Dectomax®, Zoetis Inc., USA), diluted 1:1 with sterile distilled water, was subsequently applied locally to the affected wounds as a prophylactic antiparasitic measure to reduce the risk of larval reinfestation (fly strike). Injured common buzzards and the white stork received enrofloxacin (Baytril-K 5%, Bayer Healthcare, Kiel, Germany) at 15 mg/kg/day intramuscularly (IM), together with supportive therapy (Duphalyte, Pfizer Inc., New York, NY, USA) (19–21). The tortoise and the Euphrates soft-shelled turtle received enrofloxacin at 5 mg/kg/day IM (22). In the wild goat, necrotic tissue was surgically debrided from the gluteal wound, and fluid therapy consisting of Duphalyte, 0.9% NaCl, and 5% dextrose was administered together with enrofloxacin at 2.5 mg/kg/day IM ((Baytril-K 5%, Bayer Healthcare, Kiel, Germany).

Standard biosecurity precautions were applied throughout clinical examination, wound management, and larval collection. Personnel used disposable gloves and protective clothing, instruments and working surfaces were cleaned and disinfected between cases, and routine environmental hygiene and fly-control measures were implemented to minimize cross-contamination and nosocomial fly infestation. Throughout treatment, all animals received species-appropriate supportive care, nutritional management, housing, and rehabilitation according to their clinical condition.

### 2.4 Morphological identification of larvae

For morphological examination, third-instar larvae were incised at several points using a sterile scalpel, whereas smaller specimens were punctured with a sterile pin. The specimens were cleared in 30% potassium hydroxide (KOH) until sufficient transparency was achieved, rinsed with distilled water, and air-dried. Diagnostic structures were dissected under a stereomicroscope, mounted on microscope slides using CM medium, and examined under a light microscope (Olympus CX21, Olympus Corporation, Japan).

Species identification was performed using published morphological identification keys based on the combined evaluation of diagnostic characters of third-instar larvae (23). The evaluated structures included the cephalopharyngeal skeleton, anterior and posterior spiracles, thoracic spinulation, spiracular field, and anal division, as appropriate for each taxon. Particular attention was given to the configuration of the mouthhooks, peritremes and spiracular slits, arrangement of thoracic spines, and relative positions of the posterior spiracles to distinguish closely related taxa, particularly *C. vicina* and *C. vomitoria*. Representative cephalopharyngeal skeletons and posterior spiracles of the identified species are presented in Supplementary Figure S2 (23–25).

### 2.5 Molecular analysis of COI sequences

Genomic DNA (gDNA) was extracted from 40 third-instar larvae, comprising five larvae from each of the eight clinical cases. To ensure representation of all cases and obtain suitable material for molecular confirmation, well-preserved and morphologically intact larvae with adequate tissue preservation were preferentially selected. Larval selection was therefore purposive rather than random and was intended to confirm morphological identification rather than to investigate population-level genetic variation.

Before DNA extraction, ethanol-preserved larvae were washed five times with phosphate-buffered saline (PBS). Internal tissues were digested overnight at 56 °C in 180 μL of lysis buffer, and gDNA was extracted using a commercial tissue DNA extraction kit (Thermo Fisher Scientific, Waltham, MA, USA) according to the manufacturer’s instructions. Purified DNA was stored at −20 °C until molecular analysis.

The mitochondrial cytochrome c oxidase subunit I (COI) region was amplified by polymerase chain reaction (PCR) using the conserved primers UEA7 (5′-TACAGTTGGAATAGACGTTGATAC-3′) and UEA10 (5′-TCCAATGCACTAATCTGCCATATTA-3′) (26). PCR reactions were performed in a total volume of 25 μL containing 2.5 μL of 10 × PCR buffer, 2.5 μL of 25 mM MgCl₂, 0.5 μL of dNTP mixture, 1 μL of each primer, 5 μL of template DNA, 0.125 μL of Taq DNA polymerase, and nuclease-free water to the final volume. The dNTP stock concentration was 10 mM, primer concentration was 10 pmol/μL, and Taq DNA polymerase concentration was 5 U/μL.

Thermal cycling consisted of an initial denaturation at 95 °C for 2 min, followed by 40 cycles of denaturation at 95 °C for 1 min, annealing at 52 °C for 1 min, and extension at 72 °C for 1 min, followed by a final extension at 72 °C for 7 min. PCR products were visualized by electrophoresis on a 1.4% agarose gel stained with ethidium bromide after loading 9 μL of PCR product mixed with 1 μL of TriTrack loading dye (Thermo Scientific, R1161).

Bidirectional sequencing was performed using an ABI PRISM 3100 Genetic Analyzer (Applied Biosystems, Foster City, CA, USA). Sequence chromatograms were edited and assembled using CodonCode Aligner version 11.0 (CodonCode Corporation, Dedham, MA, USA), and low-quality terminal regions were removed before subsequent analyses. Nucleotide sequence similarity was assessed using NCBI BLASTn (https://blast.ncbi.nlm.nih.gov/Blast.cgi, access date: 15.08.2026). Species assignments were evaluated by integrating morphological identification, nucleotide sequence similarity to verified GenBank reference sequences, and phylogenetic placement.

Following chromatogram editing and quality control, sequences obtained from larvae within the same clinical cases were evaluated for identity and redundancy. Eleven representative high-quality study sequences were retained for subsequent comparative and phylogenetic analyses.

### 2.6 Sequence alignment, genetic distance, and phylogenetic analysis

The 11 representative study COI sequences were analyzed together with homologous reference sequences retrieved from GenBank. The reference dataset included *L. sericata* DQ345086.1, *C. vomitoria* NC_028411.1, *C. vicina* MW266392.1, and *W. magnifica* MT027910.1. *Musca domestica* KM200723.1 was included as the outgroup. For complete or near-complete mitochondrial genome records, only the homologous COI region corresponding to the study sequences was retained. Detailed information on the reference sequences is provided in Supplementary Table S1.

Study, reference, and outgroup sequences were aligned using MUSCLE version 3.8.31 (27), as implemented in MEGA version 12.2 (28). Non-overlapping terminal positions were excluded so that all taxa were compared over the same homologous region, resulting in a final common alignment of 624 nucleotide positions. The phylogenetic dataset comprised 16 sequences in total: 11 representative study sequences, four species-specific reference sequences, and one outgroup sequence (Table 2).

**Table 2:** Species identities and accession numbers of study and reference COI sequences included in the pairwise distance analysis.

No.	Species	Accession
1	*L. sericata*	OR642793
2	*C. vomitoria*	OR642794
3	*L. sericata*	OR642795
4	*L. sericata*	OR642796
5	*L. sericata*	OR642797
6	*L. sericata*	OR642798
7	*L. sericata*	OR642799
8	*L. sericata*	OR642800
9	*C. vicina*	OR642801
10	*C. vomitoria*	OR642802
11	*W. magnifica*	OR642803
12	*L. sericata*	DQ345086.1 (Ref.)
13	*C. vomitoria*	NC_028411.1 (Ref.)
14	*C. vicina*	MW266392.1 (Ref.)
15	*W. magnifica*	MT027910.1 (Ref.)

Pairwise nucleotide divergence was calculated as uncorrected p-distance using MEGA version 12.2 (28), based on the same 624-bp common COI alignment. The pairwise-distance analysis included the 11 representative study sequences and the four verified species-specific GenBank reference sequences. The purpose of this analysis was to characterize intra- and interspecific nucleotide divergence among the focal myiasis-associated taxa and to compare the study sequences directly with their corresponding reference sequences. *M. domestica* KM200723.1 was excluded from the pairwise-distance matrix because it served solely as the phylogenetic outgroup and was not one of the focal myiasis-associated taxa. One ambiguous IUPAC nucleotide in the *C. vicina* reference sequence MW266392.1 was excluded on a pairwise basis. Pairwise distances were used descriptively and were not applied as a fixed species-delimitation threshold. The complete matrix is presented in Table 3.

**Table 3:** Pairwise uncorrected p-distances among study and reference COI sequences based on the final 624-bp common alignment.

Sequence no.	1	2	3	4	5	6	7	8	9	10	11	12	13	14	15
1	0.0000														
2	0.0849	0.0000													
3	0.0032	0.0881	0.0000												
4	0.0016	0.0849	0.0048	0.0000											
5	0.0000	0.0849	0.0032	0.0016	0.0000										
6	0.0000	0.0849	0.0032	0.0016	0.0000	0.0000									
7	0.0016	0.0865	0.0048	0.0032	0.0016	0.0016	0.0000								
8	0.0000	0.0849	0.0032	0.0016	0.0000	0.0000	0.0016	0.0000							
9	0.0913	0.0449	0.0946	0.0913	0.0913	0.0913	0.0929	0.0913	0.0000						
10	0.0849	0.0000	0.0881	0.0849	0.0849	0.0849	0.0865	0.0849	0.0449	0.0000					
11	0.1571	0.1603	0.1603	0.1554	0.1571	0.1571	0.1587	0.1571	0.1490	0.1603	0.0000				
12	0.0000	0.0849	0.0032	0.0016	0.0000	0.0000	0.0016	0.0000	0.0913	0.0849	0.1571	0.0000			
13	0.0833	0.0016	0.0865	0.0833	0.0833	0.0833	0.0849	0.0833	0.0465	0.0016	0.1603	0.0833	0.0000		
14	0.0883	0.0433	0.0915	0.0883	0.0883	0.0883	0.0899	0.0883	0.0016	0.0433	0.1493	0.0883	0.0449	0.0000	
15	0.1603	0.1635	0.1635	0.1587	0.1603	0.1603	0.1619	0.1603	0.1522	0.1635	0.0048	0.1603	0.1635	0.1525	0.0000

Pairwise nucleotide distances were calculated as uncorrected p-distances over the common 624-bp COI alignment. The matrix includes 11 representative study sequences and four verified GenBank reference sequences. Values represent the proportion of nucleotide differences per site. Reference sequences are indicated as “Ref.” The outgroup, *M. domestica* KM200723.1, was excluded because the analysis was restricted to the focal myiasis-associated taxa. One ambiguous IUPAC nucleotide in the *C. vicina* reference sequence MW266392.1 was excluded on a pairwise basis; therefore, comparisons involving this sequence were based on 623 unambiguous positions. Pairwise distances were used descriptively and not as a fixed species-delimitation threshold.

Maximum-likelihood phylogenetic analysis was performed using IQ-TREE version 3.1.3. The best-fitting nucleotide substitution model was selected automatically using ModelFinder, with candidate models ranked according to the Bayesian Information Criterion (BIC). Branch support was evaluated using 1,000 SH-like approximate likelihood ratio test (SH-aLRT) replicates and 1,000 ultrafast bootstrap (UFBoot) replicates. *M. domestica* KM200723.1 was specified as the outgroup for tree presentation, and branch lengths were expressed as substitutions per site.

## 3 Results

### 3.1 Clinical findings and distribution of cases

Traumatic myiasis was diagnosed in eight wild animals admitted between December 2020 and September 2022. The affected animals comprised four common buzzards (*B. buteo*), one white stork (*C. ciconia*), one tortoise belonging to the family Testudinidae, one wild goat (*C. aegagrus*), and one Euphrates soft-shelled turtle (*R. euphraticus*). Case-specific clinical characteristics, larval burdens, species identifications, molecular findings, and outcomes are summarized in Table 1.

All eight animals presented with open traumatic wounds. In the birds, lesions were located on the wings, whereas the two chelonian cases involved wounds beneath the shell or carapace. The wild goat presented with an open wound in the right gluteal region. Representative clinical findings in the white stork and common buzzards are shown in Supplementary Figure S1, while Figure 2 presents representative lesions in the wild goat, Euphrates soft-shelled turtle, and Testudinidae tortoise.

**Figure 2:**
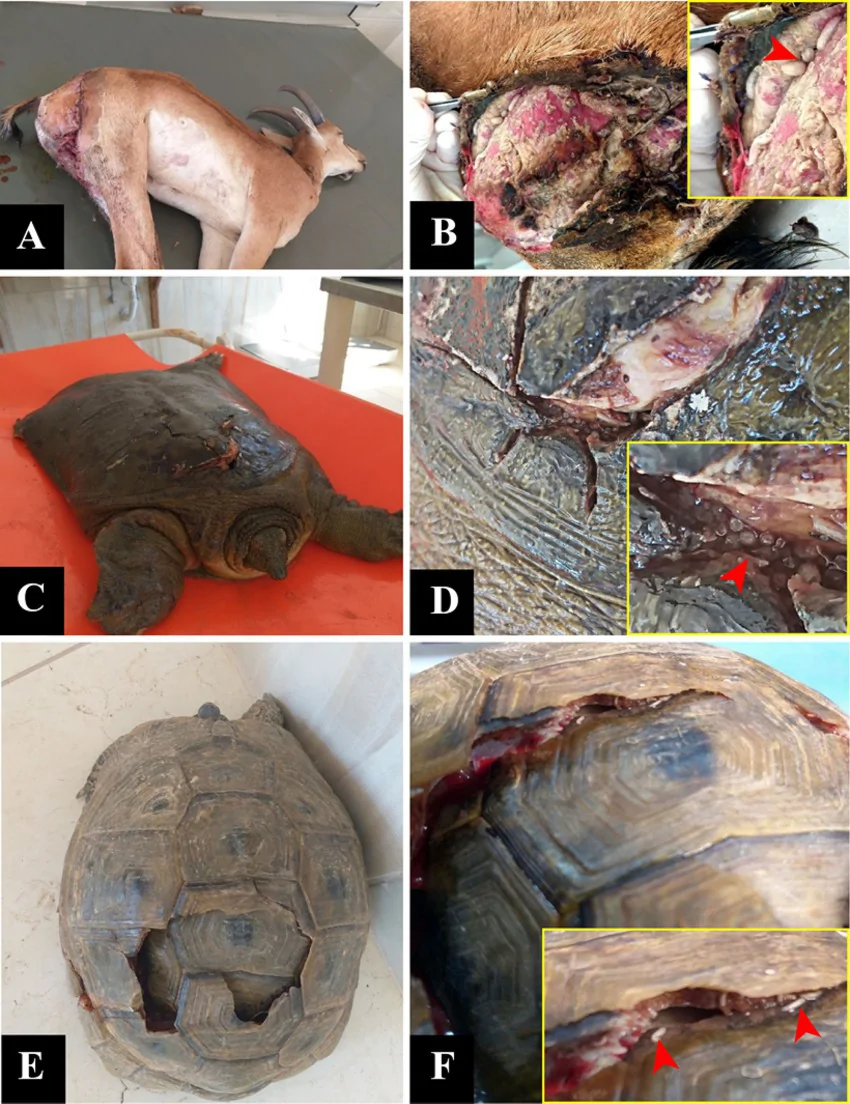
Representative macroscopic findings of traumatic myiasis in a wild goat, a Euphrates soft-shelled turtle, and a Testudinidae tortoise. **(A)** Open wound in the right gluteal region of the wild goat (*C. aegagrus*); **(B)** numerous *W. magnifica* larvae within the gluteal wound; **(C)** open wound beneath the carapace of the Euphrates soft-shelled turtle (*R. euphraticus*); **(D)** traumatic myiasis caused by *L. sericata* larvae in the wound of *R. euphraticus*; **(E)** Testudinidae tortoise with an open wound beneath the shell; **(F)** numerous *L. sericata* larvae within the wound beneath the shell. Arrows indicate larvae.

A total of 132 third-instar larvae were recovered, with individual larval burdens ranging from 5 to 42 larvae per case. Both chelonian cases were successfully rehabilitated and released. The four common buzzards recovered clinically and the white stork showed clinical improvement; however, all five birds remained under rehabilitation because of irreversible wing damage. The wild goat died despite treatment and supportive care.

### 3.2 Morphological identification of larvae

Morphological examination of all 132 third-instar larvae resulted in the identification of four dipteran species: *C. vomitoria, C. vicina, L. sericata,* and *W. magnifica*. Species assignments were based on the combined evaluation of diagnostic larval structures described in Section 2.4. Larvae identified as *C. vomitoria* showed large thoracic spines arranged separately. In contrast, *C. vicina* larvae showed smaller thoracic spines arranged in short rows, a gently curved apical portion of the mouthhooks, and posterior spiracles positioned relatively close to each other. These characters provided clear morphological differentiation between the two *Calliphora* species. *L. sericata* larvae showed a cephalopharyngeal skeleton without a sclerotized area below the posterior tip of the ventral cornua. In the spiracular field, the distance between the paired P1 papillae was approximately similar to the distance between P1 and P2.

*W. magnifica* larvae were characterized by two thick, long, and markedly curved mouthhooks, anterior spiracles with five branches, and posterior peritremes containing three spiracular slits. Together with the configuration of the cephalopharyngeal skeleton, thoracic spinulation, and anal division, these features distinguished *W. magnifica* from the calliphorid larvae examined in the present study. Representative cephalopharyngeal skeletons and posterior spiracles of the four species are presented in Supplementary Figure S2. *L. sericata* was identified in six of the eight clinical cases. *C. vomitoria* was identified in two common buzzards, *C. vicina* in one common buzzard, and *W. magnifica* in the wild goat. Mixed-species infestations occurred in two common buzzards: one contained *C. vicina* together with *C. vomitoria*, whereas the other contained *L. sericata* together with *C. vomitoria*.

### 3.3 Molecular findings and phylogenetic analysis

COI sequencing confirmed the four morphologically identified dipteran taxa. Of the 40 larvae subjected to molecular analysis, 11 representative high-quality study sequences were retained following chromatogram editing, quality control, and within-case redundancy assessment while preserving representation of each clinical case and identified species. These sequences were deposited in GenBank under accession numbers OR642793–OR642803. Sequence identities between the study sequences and their corresponding verified GenBank references ranged from 99.52 to 100.00% (Table 1). Together with four verified species-specific reference sequences and *M. domestica* KM200723.1 as the outgroup, the 11 study sequences constituted the final 16-sequence phylogenetic dataset.

Maximum-likelihood analysis of the final 624-bp common COI alignment identified TIM2 + F + I as the best-fitting nucleotide substitution model according to the Bayesian Information Criterion (BIC = 3699.926). The study sequences clustered with their corresponding reference taxa with strong species-level support (Figure 3). *W. magnifica* OR642803 grouped with MT027910.1 with maximal support (SH-aLRT/UFBoot = 100/100). *C. vicina* OR642801 clustered with MW266392.1 with strong support (96.5/96), while the two *C. vomitoria* study sequences clustered with NC_028411.1 with strong support (98.7/98). The *L. sericata* study sequences formed a strongly supported clade with DQ345086.1 (98.3/99).

**Figure 3:**
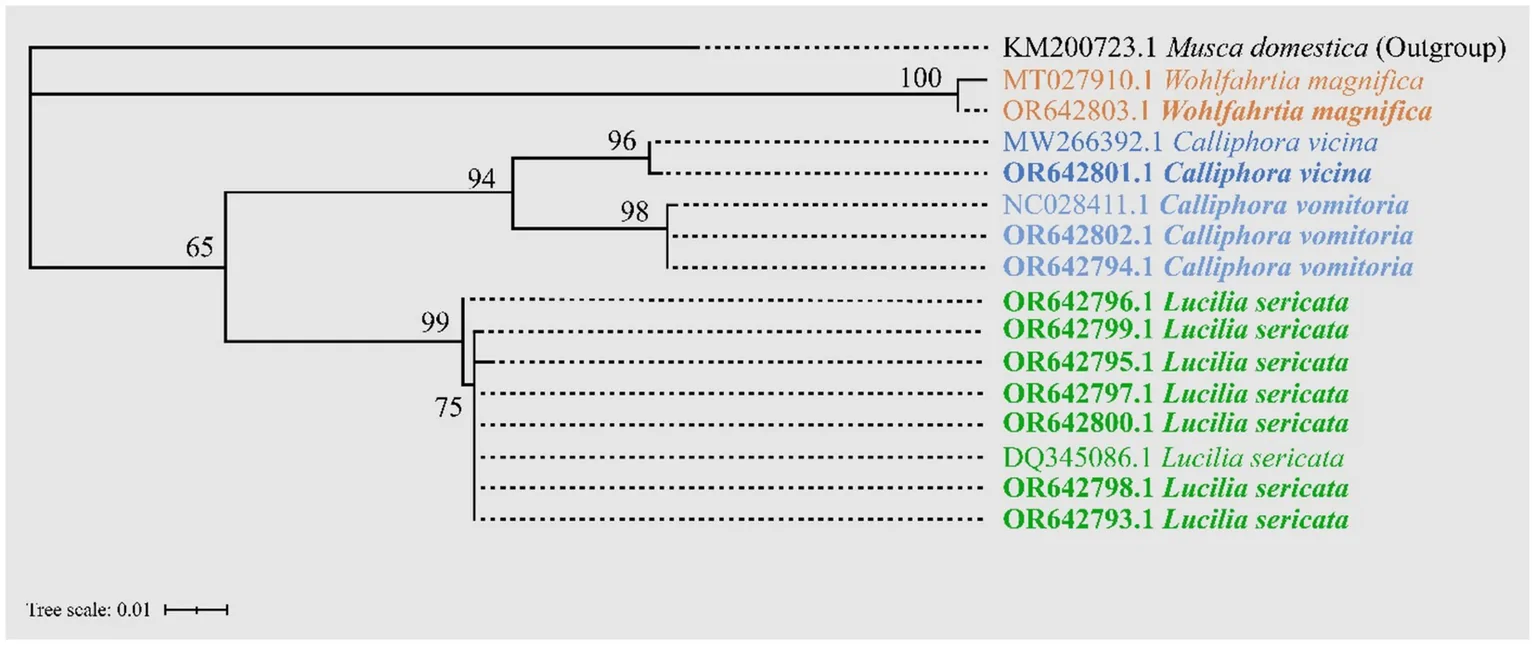
Maximum-likelihood phylogenetic tree based on mitochondrial COI sequences. The tree was inferred from a 624-bp common homologous COI alignment comprising 16 sequences using IQ-TREE version 3.1.3. The TIM2 + F + I nucleotide substitution model was selected by ModelFinder according to the Bayesian Information Criterion. Numbers shown at the nodes represent ultrafast bootstrap (UFBoot) support percentages based on 1,000 replicates; corresponding SH-aLRT/UFBoot support values for the principal species-level clades are reported in the Results. Sequences generated in the present study (GenBank accessions OR642793–OR642803) are shown in bold. *M. domestica* KM200723.1 was used as the outgroup. The scale bar indicates substitutions per site.

Pairwise uncorrected p-distance analysis showed very low within-species divergence relative to between-species divergence (Table 3). Among the seven *L. sericata* study sequences, the mean p-distance was 0.0018 (range, 0.0000–0.0048). OR642793, OR642797, OR642798, and OR642800 were identical to the *L. sericata* reference DQ345086.1 across the analyzed 624-bp region (p-distance = 0.0000). The two *C. vomitoria* study sequences, OR642794 and OR642802, were identical to each other and differed from NC_028411.1 by 0.0016. *C. vicina* OR642801 differed from MW266392.1 by 0.0016, whereas *W. magnifica* OR642803 differed from MT027910.1 by 0.0048.

Between the study sequences, *C. vomitoria* differed from *C. vicina* by 0.0449. Pairwise distances between the *L. sericata* and *Calliphora* study sequences ranged from 0.0849 to 0.0946, whereas *W. magnifica* OR642803 differed from the other study sequences by 0.1490–0.1603. The complete study-and-reference pairwise-distance matrix is presented in Table 3.

## 4 Discussion

Traumatic myiasis is generally regarded as an opportunistic parasitic condition associated with pre-existing wounds, tissue necrosis, impaired mobility, or poor body condition (29). In the present series, traumatic open lesions were present in all affected animals, supporting the role of tissue injury as an important predisposing condition for larval colonization. The predominance of wing injuries among the avian cases and wounds beneath the shell or carapace in the chelonians further illustrates how exposed or difficult-to-protect lesions may provide suitable sites for infestation. These observations are consistent with previous reports identifying traumatic injury and impaired mobility as important predisposing factors for myiasis in wildlife (3, 6). Nevertheless, because this was a small opportunistic case series, the findings should be interpreted descriptively rather than as estimates of risk or occurrence.

The ecological setting of Diyarbakır is also relevant when interpreting the temporal distribution of the cases. Previous entomological surveillance in the region demonstrated seasonal activity of myiasis-associated flies, including *L. sericata, C. vicina,* and *C. vomitoria*, with greater adult activity during warmer months (18). Most cases in the present series likewise occurred between April and September. The single case recorded in December should therefore be interpreted cautiously and may reflect case-specific factors such as prolonged wound exposure, local microclimatic conditions, or short-term weather variation rather than a consistent winter pattern.

The frequent detection of *L. sericata* in this series is consistent with its recognized role as a widespread facultative agent of traumatic myiasis in birds and other vertebrates, particularly in association with exposed wounds (5, 8, 18). Reports of avian myiasis also support its importance as an opportunistic wound-colonizing species (5). However, the small number of cases and the admission-based nature of the present study do not permit conclusions regarding its prevalence or ecological dominance in southeastern Turkey. The occurrence of mixed-species infestations in two common buzzards is also consistent with previous reports showing that traumatic wounds may be colonized by more than one myiasis-associated fly species (8, 30). Such coexistence may reflect overlapping attraction to exposed or necrotic tissues or sequential colonization of the same wound. The present data, however, do not allow determination of whether mixed infestation altered larval burden, wound progression, treatment response, or prognosis.

The occurrence of *W. magnifica* in the wild goat is clinically relevant because this species is an obligate myiasis-producing sarcophagid capable of causing extensive tissue damage. *W. magnifica* has been documented in domestic mammals, including goats, and in other mammalian hosts (31–34). To the best of our knowledge, this study provides the first worldwide records of traumatic myiasis in the wild goat (Capra aegagrus). In the present case, infestation involved a deep gluteal wound and was accompanied by progressive clinical deterioration. Although the animal subsequently died, the outcome should not be attributed solely to myiasis because severe underlying trauma and deterioration of the general condition were also present.

Traumatic myiasis in the Euphrates soft-shelled turtle (*R. euphraticus*) represents another noteworthy observation. This species is restricted to the Euphrates–Tigris basin and is classified as Endangered, with habitat alteration and fragmentation constituting important conservation concerns (35, 36). Traumatic myiasis has previously been reported in other chelonians, including Hermann’s tortoise (37), but, to the best of our knowledge, it has not previously been documented in *R. euphraticus*. The present case therefore extends the documented host range of traumatic myiasis in chelonians and indicates that wound-associated larval infestation should be considered during rehabilitation of injured *R. euphraticus*. A single case, however, does not allow any inference regarding population-level conservation impact.

The molecular analyses provided independent support for the morphological identifications. Concordance among morphological characters, sequence similarity, phylogenetic placement, and pairwise nucleotide divergence strengthened the species-level assignments. The comparatively low support for some internal branches within the *L. sericata* clade is consistent with the very low intraspecific COI divergence observed among these sequences and does not affect their species-level placement (38). Importantly, pairwise distances were interpreted descriptively and were not used as an independent species-delimitation threshold. The combined morphological and molecular approach therefore provided complementary evidence rather than relying on a single identification criterion.

Clinical outcomes should be interpreted in the context of individualized multimodal wound management and the severity of the underlying traumatic injuries. Although the two chelonian cases recovered and were released, the birds remained under rehabilitation because of irreversible wing damage, whereas the wild goat died following progressive deterioration. Because the study was observational, treatment was not standardized for comparative efficacy assessment, and no untreated or alternative-treatment group was available, no causal conclusions regarding the effectiveness of doramectin, antimicrobial therapy, or the overall treatment regimen can be drawn.

This study has several limitations. The number of cases was small and all animals were admitted to a single wildlife rehabilitation center; therefore, the findings cannot be generalized to free-ranging wildlife populations or used to estimate prevalence. Sampling was opportunistic and admission-based, potentially favoring animals with more severe trauma or more advanced infestations. Larvae selected for molecular analysis were chosen purposively for species confirmation, and the molecular component was not designed to assess population-level genetic diversity. Environmental and microclimatic variables were not systematically recorded, limiting broader ecological interpretation. In addition, one tortoise could not be identified beyond the family level. Despite these limitations, the combined clinical, morphological, and molecular documentation provides useful descriptive information on traumatic myiasis in injured wildlife from southeastern Turkey.

## 5 Conclusion

This study provides integrated clinical, morphological, and molecular documentation of traumatic myiasis in wild animals admitted to a rehabilitation center in southeastern Turkey. Concordance between morphological characters and COI-based molecular analyses supported reliable species-level identification of the causative larvae and documented uncommon host associations, including traumatic myiasis in *R. euphraticus*. Although the findings are based on a small, opportunistic single-center case series, they contribute to the limited knowledge of wildlife-associated traumatic myiasis and emphasize the importance of accurate larval identification during the clinical management of injured wildlife.

## Data Availability

The datasets presented in this study can be found in online repositories. The names of the repository/repositories and accession number(s) can be found in the article/Supplementary material.

## References

[1] Hall MJR Wall RL Stevens JR. Traumatic myiasis: a neglected disease in a changing world. Annu Rev Entomol. (2016) 61:159–76. doi: 10.1146/annurev-ento-010715-023655 26667275

[2] Francesconi F Lupi O. Myiasis. Clin Microbiol Rev. (2012) 25:79–105. doi: 10.1128/CMR.00010-11, 22232372 PMC3255963

[3] Bugelli V Tarozzi I Galante N Bortolini S Franceschetti L. Review on forensic importance of myiasis: focus on medicolegal issues on post-mortem interval estimation and neglect evaluation. Leg Med (Tokyo). (2023) 63:102263. doi: 10.1016/j.legalmed.2023.102263, 37126932

[4] Jallow BJJ Gassara G Bajinka O Luo Y Liu M Cai J . Human myiasis in sub-Saharan Africa: a systematic review. PLoS Negl Trop Dis. (2024) 18:e0012027. doi: 10.1371/journal.pntd.0012027, 38547087 PMC10977789

[5] Pezzi M Krcmar S Mendicino F Carlomagno F Bonelli D Scapoli C . *Lucilia sericata* (Diptera: Calliphoridae) as agent of myiasis in a goose in Italy and a review of myiasis by this species in birds. Insects. (2022) 13:542. doi: 10.3390/insects13060542, 35735879 PMC9224840

[6] Tompkins DM Dunn AM Smith MJ Telfer S. Wildlife diseases: from individuals to ecosystems. J Anim Ecol. (2011) 80:19–38. doi: 10.1111/j.1365-2656.2010.01742.x, 20735792

[7] Alikhan M Al-Ghamdi K Zahrani F Khater E Allam A. Prevalence and salient morphological features of myiasis causing dipteran flies in Jeddah, Saudi Arabia. Biosci Biotechnol Res Asia. (2018) 15:101–9. doi: 10.13005/bbra/2612

[8] Obanda V Ndambiri EM Kingori E Gakuya F Lwande OW Alasaad S. Traumatic myiasis in free-ranging eland, reported from Kenya. Parasit Vectors. (2013) 6:89. doi: 10.1186/1756-3305-6-89, 23566876 PMC3636081

[9] Salimi M Goodarzi D Karimfar M Edalat H. Human urogenital myiasis caused by *Lucilia sericata* (Diptera: Calliphoridae) and *Wohlfahrtia magnifica* (Diptera: Sarcophagidae) in Markazi Province of Iran. Iran J Arthropod Borne Dis. (2010) 4:72–6. PMC3385545 22808392

[10] Ojeyinka O Omaghomi T. Wildlife as sentinels for emerging zoonotic diseases: a review of surveillance systems in the USA. World J Adv Res Rev. (2024) 21:768–76. doi: 10.30574/wjarr.2024.21.3.0773

[11] Wall R. Ovine cutaneous myiasis: effects on production and control. Vet Parasitol. (2012) 189:44–51. doi: 10.1016/j.vetpar.2012.03.031, 22541796

[12] Hosni EM Nasser MG Al-Ashaal SA Rady MH Kenawy MA. Modeling current and future global distribution of *Chrysomya bezziana* under changing climate. Sci Rep. (2020) 10:4947. doi: 10.1038/s41598-020-61962-8, 32188920 PMC7080715

[13] Stevens J Wall R. Genetic relationships between blowflies (*Calliphoridae*) of forensic importance. Forensic Sci Int. (2001) 120:116–23. doi: 10.1016/S0379-0738(01)00417-0, 11457618

[14] Ross AG Papier K Luceres-Catubig R Chau TN Inobaya MT Ng SK. Poverty, dietary intake, intestinal parasites, and nutritional status among school-age children in the rural Philippines. Trop Med Infect Dis. (2017) 2:49. doi: 10.3390/tropicalmed2040049, 30270906 PMC6082057

[15] Feldmeier H. "Ectoparasites and arthropod vectors: ectoparasite infestations". In: Heggenhougen HK, editor. International Encyclopedia of Public Health. Oxford: Academic Press (2008). p. 282–94.

[16] Scholl PJ Colwell DD Cepeda-Palacios R. "Chapter 19 – Myiasis (*Muscoidea*, *Oestroidea*)". In: Mullen GR Durden LA, editors. Medical and Veterinary Entomology, 3rd Edn. London: Academic Press (2019). p. 383–419.

[17] Myiasis. Veterinary Ectoparasites: Biology, Pathology and Control. (2001). p. 114–42. Oxford, United Kingdom: Blackwell Science.

[18] Ipek D Saki C. Seasonal distributions of external myiasis flies determined in Diyarbakır Province. J Kafkas Univ Vet Fak. (2009) 17:469–75. doi: 10.9775/kvfd.2011.4038

[19] Harrenstien L Tell L Dvm R Needham BA Brandt BS Brondos BA . Disposition of enrofloxacin in red-tailed hawks (*Buteo jamaicensis*) and great horned owls (*Bubo virginianus*) after a single oral, intramuscular, or intravenous dose. J Avian Med Surg. (2009) 14:228–36. doi: 10.1647/1082-6742(2000)014[0228:DOEIRT]2.0.CO;2

[20] Lacasse C. Falconiformes (Falcons, Hawks, Eagles, Kites, Harriers, Buzzards, Ospreys, Caracaras, Secretary Birds, Old World and New World Vultures). St. Louis, MO, United States: Elsevier Saunders. (2015). p. 127–42.

[21] Poeta P Sargo R Valente J Prada J Silva F Felício C . *Serratia marcescens* discospondylitis in a white stork (*Ciconia ciconia*). SOJ Microbiol Infect Dis. (2016) 4:1–5. doi: 10.15226/sojmid/4/3/00155

[22] Raphael BL Papich M Cook RA. Pharmacokinetics of enrofloxacin after a single intramuscular injection in Indian star tortoises (*Geochelone elegans*). J Zoo Wildl Med. (1994) 25:88–94.

[23] Szpila K Madra A Jarmusz M Matuszewski S. Flesh flies (Diptera: *Sarcophagidae*) colonising large carcasses in Central Europe. Parasitol Res. (2015) 114:2341–8. doi: 10.1007/s00436-015-4431-1, 25876045 PMC4430587

[24] Szpila K Richet R Pape T. Third instar larvae of flesh flies (Diptera: *Sarcophagidae*) of forensic importance--critical review of characters and key for European species. Parasitol Res. (2015) 114:2279–89. doi: 10.1007/s00436-015-4421-3, 25823900 PMC4430590

[25] Szpila K. Key for the Identification of Third Instars of European Blowflies (Diptera: Calliphoridae) of Forensic Importance. Dordrecht, Netherlands: Springer. (2010). p. 43–56.

[26] Otranto D Traversa D Guida B Tarsitano E Fiorente P Stevens JR. Molecular characterization of the mitochondrial cytochrome oxidase I gene of Oestridae species causing obligate myiasis. Med Vet Entomol. (2003) 17:307–15. doi: 10.1046/j.1365-2915.2003.00442.x, 12941016

[27] Edgar RC. MUSCLE: multiple sequence alignment with high accuracy and high throughput. Nucleic Acids Res. (2004) 32:1792–7. doi: 10.1093/nar/gkh340, 15034147 PMC390337

[28] Kumar S Stecher G Suleski M Sanderford M Sharma S Tamura K. MEGA12: molecular evolutionary genetic analysis version 12 for adaptive and green computing. Mol Biol Evol. (2024) 41:msae263. doi: 10.1093/molbev/msae263, 39708372 PMC11683415

[29] Sunny B Sulthana L James A Sivakumar T. Maggot infestation: various treatment modalities. J Am Coll Clin Wound Spec. (2016) 8:51–3. doi: 10.1016/j.jccw.2018.03.002, 30276127 PMC6161638

[30] Farkas R Szántó Z Hall M. Traumatic myiasis of geese in Hungary. Vet Parasitol. (2001) 95:45–52. doi: 10.1016/S0304-4017(00)00409-X, 11163697

[31] Gunn A Bird J. The ability of the blowflies *Calliphora vomitoria* (Linnaeus), *Calliphora vicina* (rob-Desvoidy) and *Lucilia sericata* (Meigen) (Diptera: Calliphoridae) and the muscid flies *Muscina stabulans* (fallen) and *Muscina prolapsa* (Harris) (Diptera: Muscidae) to colonise buried remains. Forensic Sci Int. (2011) 207:198–204. doi: 10.1016/j.forsciint.2010.10.008, 21071161

[32] Jia Z Hasi S Zhan D Vogl C Burger PA. Transcriptomic profiling of different developmental stages reveals parasitic strategies of *Wohlfahrtia magnifica*, a myiasis-causing flesh fly. BMC Genomics. (2024) 25:111. doi: 10.1186/s12864-023-09949-3, 38297211 PMC10829477

[33] Bouquet J. Perineal myiasis caused by *Wohlfahrtia magnifica* in mares in Central France. Vet Rec Case Rep. (2024) 12:e787. doi: 10.1002/vrc2.787

[34] Beyhan YE Yılmaz H Taş Cengiz Z Ayral A. A case of aural myiasis caused by *Wohlfahrtia magnifica* in a child in Turkey. Turk Bull Hyg Exp Biol. (2017) 74:347–50. doi: 10.5505/turkhijyen.2017.09825

[35] Eren G Açıcı M Gürler AT Bölükbaş CS Umur Ş. A case of preputial myiasis caused by *Wohlfahrtia magnifica* (Diptera: Sarcophagidae) in a goat (*Capra hircus*). Van Vet J. (2022) 33:42–5. doi: 10.36483/vanvetj.1022698

[36] Fois F Mereu Piras P Cappai S Vanin S Maistrello L Deiana AM . "Myiasis by *Wohlfahrtia magnifica* (Diptera: Sarcophagidae) in different mammals in Sardinia". In: XXVII National Congress of the Italian Society of Parasitology (SoIPa); 2012/06/26. Alghero, Italy: (2012). p. 107.

[37] Taskavak E Atatür M Ghaffari H Meylan P. Rafetuseuphraticus (Daudin 1801)–Euphrates Softshell Turtle. Arlington, VT, United States: Chelonian Research Foundation. (2016). p. 98:1–11.

[38] Wang X Zhong Z Shan Y Guo Q Cheng Z Song X . Old World screwworm myiasis: first report of *Chrysomya bezziana* in Chinese Milu (*Elaphurus davidianus*). Acta Trop. (2026) 273:107938. doi: 10.1016/j.actatropica.2025.107938, 41360210

