# Supplementary material for: Traumatic myiasis in wild animals admitted to a rehabilitation center in southeastern Turkey
Source: Front Vet Sci. 2026 Oct 6;13:1892985. doi: 10.3389/fvets.2026.1892985 (PMC13680164; doi:10.3389/fvets.2026.1892985)
Supplement: Supplementary file 1 [file Data_Sheet_1.docx]

**SUPPLEMENTARY TABLE**

**Supplementary Table S1. GenBank reference sequences used in the revised COI phylogenetic analysis. Reference sequences were selected to represent the four larval species identified morphologically and molecularly in the present study. For complete mitochondrial genome records, only the homologous COI segment overlapping the study sequences was retained. All study and reference sequences were aligned and trimmed to a common 624-bp homologous COI region before maximum-likelihood phylogenetic analysis. Musca domestica KM200723.1 was included as the outgroup.**

**Supplementary Table S1. GenBank reference sequences used in the revised COI phylogenetic analysis.**

| **Species** | **GenBank accession** | **Sequence type** | **Reference/source** | **Region used in present analysis** | **Role** |
| --- | --- | --- | --- | --- | --- |
| *Lucilia sericata* | DQ345086.1 | COI gene, partial cds; mitochondrial | GenBank record; also included as a Chinese *L. sericata* COI reference in subsequent phylogenetic studies | Homologous portion included in the final 624-bp alignment | Species reference |
| *Calliphora vomitoria* | NC_028411.1 | Complete mitochondrial genome | NCBI RefSeq/GenBank mitochondrial genome record; reported in subsequent mitogenomic datasets as a *C. vomitoria* reference | Homologous COI segment corresponding to the study sequences | Species reference |
| *Calliphora vicina* | MW266392.1 | Complete mitochondrial genome | Palevich N, Carvalho L, Maclean P. Characterization of the complete mitochondrial genome of the New Zealand parasitic blowfly *Calliphora vicina*. *Mitochondrial DNA Part B*. 2021 | Homologous COI segment corresponding to OR642801 | Species reference |
| *Wohlfahrtia magnifica* | MT027910.1 | COX1 gene, partial cds; mitochondrial | GenBank COX1 reference record; isolate 2 | Homologous portion overlapping OR642803 and the final 624-bp alignment | Species reference |
| *Musca domestica* | KM200723.1 | Complete mitochondrial genome | Li X, Wang Y, Su S, Yang D. The complete mitochondrial genomes of *Musca domestica* and *Scathophaga stercoraria*. *Mitochondrial DNA*. 2016;27:1435–1436 | Homologous COI segment corresponding to the final 624-bp alignment | Outgroup |

Supplementary Figures

Supplementary Figures S1 and S2 provide representative clinical and morphological findings from the traumatic myiasis cases included in this study. Figure S1 illustrates representative wound and larval infestation findings in affected wild birds, whereas Figure S2 presents the principal morphological structures of third-instar larvae used for species identification prior to molecular confirmation.

Figure S1. Representative clinical findings of traumatic myiasis in a white stork (*Ciconia ciconia*) and common buzzard (*Buteo buteo*). (A) White stork with a traumatic wing wound; (B) wing wound of the white stork infested with numerous *Lucilia* *sericata* larvae; (C) common buzzard showing a depressed clinical condition; (D) ventral view of the wing wound of the common buzzard showing larvae of different species; (E) fractured wing of the common buzzard with numerous *L.* *sericata* larvae within the wound

**Figure S2.** Diagnostic morphological structures of third-instar larvae of *Calliphora vomitoria*, *Calliphora* *vicina*, *Lucilia* *sericata*, and *Wohlfahrtia* *magnifica*. (A) *C. vomitoria*, cephalopharyngeal skeleton, lateral view; (B) *C. vomitoria*, posterior spiracles; (C) *C. vicina*, cephalopharyngeal skeleton, lateral view; (D) *C. vicina*, posterior spiracles; (E) *L. sericata*, cephalopharyngeal skeleton, lateral view; (F) *L. sericata*, posterior spiracles; (G) *W. magnifica*, cephalopharyngeal skeleton, lateral view; (H) *W. m*agnifica, posterior spiracles. The cephalopharyngeal skeleton and posterior spiracles were the principal diagnostic structures used for morphological species identification prior to molecular confirmation.
